# Supplementary material for: Semi-automated socio-anthropologic analysis of the medical discourse on rheumatoid arthritis: Potential impact on public health
Source: PLoS One. 2022 Dec 29;17(12):e0279632. doi: 10.1371/journal.pone.0279632 (PMC9799325; doi:10.1371/journal.pone.0279632)
Supplement: S1 File — (DOCX) [file pone.0279632.s004.docx]

**Semi-automatic socio-anthropologic analysis of the *medical discourse* on rheumatoid arthritis:**

**potential impact in public health**

**Supporting Information**

# Christine Nardini^1*^,Lucia Candelise^2,3*^, Mauro Turrini^4*^, Olga Addimanda^5^

^1^ Consiglio Nazionale delle Ricerche, Istituto per le Applicazioni del Calcolo "Mauro Picone", Roma, IT

^2^ ISS, Istitut Sciences Sociales, Université de Lausanne, CH

^3^ CEPED, Centre Population et Développement, Université de Paris, FR

^4^Institute of Public Goods and Policies (IPP), Spanish National Research Council (CSIC), Madrid - ES

^5^UOC Medicina Interna ad Indirizzo Reumatologico, Ospedale Maggiore, AUSL Bologna, IT

*To whom correspondence should be addressed: [christine.nardini@cnr.it](mailto:christine.nardini@cnr.it), [lucia.candelise@unil.ch](mailto:lucia.candelise@unil.ch),

[mauro.turrini@cchs.csic.es](mailto:mauro.turrini@cchs.csic.es)

***Key-words:*** non-communicable diseases; non-pharmacological therapies; chronic inflammation; rheumatoid arthritis; health policy; medical socio-anthropology; machine learning; Sustainable Development Goals.

**Abstract**

**BACKGROUND** The debilitating effects of non-communicable diseases (NCDs) and the accompanying chronic inflammation, represent a significant obstacle for the sustainability of our development, with efforts being spread worldwide to contrast NCDs’ diffusion, as per the United Nations Sustainable Development Goals (SDG 3). In fact, despite efforts of variable intensities in numerous directions (from innovations in biotechnology to lifestyle modifications), NCDs’ incidence remains pandemic. The present work wants to contribute to this major concern with a specific focus on the fragmentation of the medical approaches, via an interdisciplinary analysis of the *medical discourse*, i.e. the heterogenous reporting that biomedical scientific literature uses to describe the anti-inflammatory therapeutic landscape in NCDs. The aim is to better capture the roots of this compartmentalization and the power relations existing among three segregated *pharmacological*, *experimental* and *unstandardized* biomedical approaches, to ultimately empower collaboration beyond medical specialties and possibly untap a more ample and effective reservoir of integrated therapeutic opportunities.

**METHOD** Using as exemplar disease rheumatoid arthritis (RA), twenty-eight articles were manually translated each into a nine-dimensional categorical variable of medical socio-anthropological relevance, relating in particular (but not only) to *legitimacy*, *temporality* and *spatialization*. This digitalized picture (9 x 28 table) of the medical discourse was further analyzed by simple automatic learning approaches to identify differences and highlight commonalities among the biomedical categories.

**RESULTS** Interpretation of these results gives original insights including the suggestion to: empower scientific communication between *unstandardized* approaches and basic biology; promote non-pharmacological therapies repurposing to enhance robustness of *experimental* approaches; align the spatial representation of diseases and therapies in *pharmacology* to effectively embrace the *systemic* approach promoted by modern personalized and preventive medicines. We hope this original work may expand and foster interdisciplinarity among public health stakeholders, ultimately contributing to the achievement of SGD3.

**Literature additional filtering criteria**

The queries on Pubmed were run on February 4^th^ 2020. Before analyzing the output articles additional filtering has been applied on titles alone in order to refine the selection. The first filtering is a semi-automatic step, the second is manual.

Semi-automatic filtering to remove the following items (color coded in Supplementary Table 1):

- Non RA: reviews not on RA
- Non English: articles in other languages
- Non appropriate: different topic
- Too specific: RA with specific comorbidity; case reports; non systemic approach to RA (i.e. specific joints only)
- Too general: relevant for RA also, but not specific to RA (green)
- Included: the article already falls in another category, we kept it in the most appropriate one
- Misclassified: belongs to another category, where it is not present (orange)
- Older version: when two versions are available we keep the most recent

Second manual curation:

- Identification title-wise of the most general articles, coherent with the subsection of interest. This, in particular for the most abundant category of drug therapy anti-inflammatory, leads to the arguable exclusion (*too specific*) of any article focusing on any broad subset of drugs (DMARDs, chemotherapics etc.)
- Identification where possible of time series, mostly *drug therapy anti-inflammatory,* *dietary supplement,* a*nti-microbial*. The former for example traces back the evolution of therapy from the introduction of biologics onwards, and the latter morphs into the more general *gastrointestinal microbiome* upon discovery of its relevance in autoimmunity.
- Filtering by availability (availability of the online article in general, and open access afterwards)
- Cochrane reviews are preferred when offered by PubMed ranking by all above criteria.
- Control on specific bias: There exist ample controversy against the usage of sham acupuncture as a mean to include blinding in random control trials (RCT) for acupuncture [1,2]. Opponent to this forceful blinding claim that all points on the body surface are to some extent sensitive to the needle stimulation and therefore comparison is meaningless/inappropriate. Beyond the controversy, there exist internationally standardized [3] statistical design to model situations where placebo is not possible (this, incidentally, includes degenerative diseases like RA): *non-inferiority clinical trial*s, that compare the gold standard (pharmacological therapy for RA) with the same therapy plus an additional approach, acupuncture in this case. Given that the two automatically selected papers [4] and [5] include sham acupuncture and/or exclude non-inferiority trials, in order to offer a more complete analysis of the medical discourse we added the article by Wang et al. [6] published on the same year as [5] and analyzing the same data (7 out of 8 total trials are the same), including active control trials and analyzing them separately from RCT.

### **Variables – additional details**

The identification of the variables relevant to handle the analysis followed several steps. First, upon careful study of the available literature, it was considered appropriate to identify categories from the areas of medical sociology [7] and anthropology [8], as well as some general attributes, to capture the essence of the *medical discourse* expressed in the selected material. Table 1 of the main manuscript reports this step in the column *Area*. Second, intense discussion identified the categories that best captured in socio-anthropological terms the observed ample variance among the articles, namely the *Temporality* [9], *Legitimacy* [10,11] and *Spatialization* of the approaches [9,12], backed by the additional attributes: *Typology* of the article, Diagnostic *Criterion*, *Background* of the authors, *Date* of Publication, *Geography* (column *Variables*). Third, in order to go beyond the study of individual articles and make the process of analysis more manageable, variables that were not inherently categorical, where discretized (column referring to the mathematical *Nature* of the variable). This additional step implied a standardization of the values of each variable (column *Values*). Values were assessed by a sociologist and an anthropologist for the socio-anthropological variables each on a subset of articles, and by a systems biologist for the general variables and for inference on the FMT study, using the controlled MeSH vocabulary [13], where appropriate. Finally, variables were observed for the dimensionality of their level (column *#Levels*), with variables characterized by a number of levels comparable to the sample size (twenty-eight) being further processed by *textual analysis*, and the other by *automatic learning* (see Methods). Follows the definitions of the nine selected variables.

*1. Temporality.* The two main sources of scientific soundness – experiential knowledge and technoscientific innovation *–* are inscribed within two opposing temporal polarities: past and future [9]. *Experiential* knowledge is associated with established practices, that have been adopted and proven effective within comparable clinical frames for a sufficiently long time in the (recent) past. Technoscientific *innovation* is associated to new tendencies within biomedical research. In such cases, a treatment is deemed promising in light of the results it has produced in basic research, *in vitro*, *in vivo* or in early stages of clinical validation. An exception, the very peculiar and unique article on electromagnetism [14], forced us to add an intermediate level: *syncretism*.

2. *Legitimacy*: describes the relationship of a therapy considered vis-à-vis the dominant pharmacological approach [10]**.** This category is intended to capture whether a therapeutic approach proposes itself as an alternative or a complementary remedy. We chose to use these two categories that refer to the now popular definition given in the 1990s by the US National Institutes of Health [15], where the definition of Complementary and Alternative Medicine (CAM) was coined. In our work we use the terms *complementary* or *alternative* to indicate the *relative* relationship that a given treatment has with any other treatment, not to biomedicine alone. Generally, we speak of complementary or alternative *with respect to* biomedicine. However, in some cases this relationship refers to different treatments [16]. In few cases [5,6] it was necessary to introduce an additional intermediate value for the dual/ambiguous relationship with other medical approaches, using *alt-complementary*. The literature on anti-inflammatory drugs represents the *reference* and is labeled as such.

3. S*patialization of the disease’s etiology*. This variable analyzes in which region of the body the medical discourse locates the disease’s origin (etiology) [12]. Modern medicine generally makes the disease evolve from an original place, the *lesion*, located at a very precise point in the depth of the organs and verifiable through autopsy [17]. Some authors [18] advanced the idea that in contemporary biomedicine the *lesion* is represented at the molecular level, as the interaction between enzymes, proteins and other biochemical or cellular elements. This is the case of the dominant PHA approach to RA, according to which this condition descends from a molecular, immunological abnormality (partly genetic and partly environmental) that leads to an autoimmune response, inflammation, senescence, and, finally, to the joints damage. Other approaches, however, adopt a more holistic vision of the body and, accordingly, interpret pathology as a more general condition, involving interactions between different organs, or relationships with the outside (for instance food). Traditional approaches usually tend to be holistic, yet, within the biomedical world, systemic approaches to the body can also be found, as it is the case for the role of the GI microbiome. With regard to body geographies, the distinction was made between *systemic* approaches, which conceive the disease as a widespread phenomenon in the organism and arising from the interaction of several organs and functional systems, or *localized*, in the case of approaches that understand the disease as the result of a lesion located in a specific area.

4. *Spatialization of the disease’s therapy.* In our pool of articles we observed that the same rationale described above could be applied to the spatialization of the therapy, as the two (spatialization of the etiology *or* of the disease) did not necessarily overlap. A therapy is systemic when it considers the healing processes as involving the whole organism, or the interaction with the environment (as it is the case for nutrition), or different regions of the body (as it is the case for VNS or dysbiosis), or holistic body descriptors (such as *meridians* in Traditional Chinese Medicine). When the perceptions of the disease and of the therapy spatially coincide (i.e. the values of the two spatialization variables overlap, they are both *local* or both *systemic*), we define them as *coherent* and *incoherent* otherwise.

*5. Typology:* This criterion has emerged with force from the manual curation of the articles. Despite all articles belonging to the biomedical scientific literature (all responding to the definition of ‘review’ as MeSH search term) they consider a wide set of different articles and adopt different analytical approaches. In particular, we chose to make a distinction between *systematic review* and *overview*. *Systematic reviews* are committed to analyze the impact of a specific treatment based on human clinical trials selected through specific criteria and they include (but are not limited to) statistical *meta-analyses*, whereas *overviews* collect in a less systematic way different types of scientific publications (clinical trials, but also *in vitro* or animal studies) on the basis of the authors' knowledge of the state-of-the-art with the aim to offer insights about a particular technique or research branch.

6. Diagnostic *Criterion.* This refers to the description of the diagnosis and disease progression, against a list of standardized parameters. These are the definition regularly updated by the American Congress of Rheumatology (ACR) and European League for Arthritis Rheumatoid, EULAR, often also referred jointly as ACR/EULAR [19], relying on clinical, molecular as well as functional parameters assessed by patients and doctors (using in particular the Disease Activity Score, DAS28 to objectively assess the number of affected joints [20]), and the OMERACT ([21] consensus-derived collection of outcomes and instruments to measure a consistent set of clinical endpoints in RA, with a particular focus on the damage assessed by ultrasound). Finally, ROM (range of motion score, to measure the distance and direction that a joint can stretch) was used exclusively in the review on massage [22] In one case only two indexes are used jointly (ACR OMERACT, ultrasound therapy [23])

7. *Background* of the authors, using as proxy the MeSH term corresponding to the specialty of the Department or (where absent) of the Institute of affiliation.

8. *Date of Publication*, by year.

9. *Geography* using the State of the main author's institution of affiliation. This has been used as a loose proxy for the cultural context the article emerges from.

**Data representation**


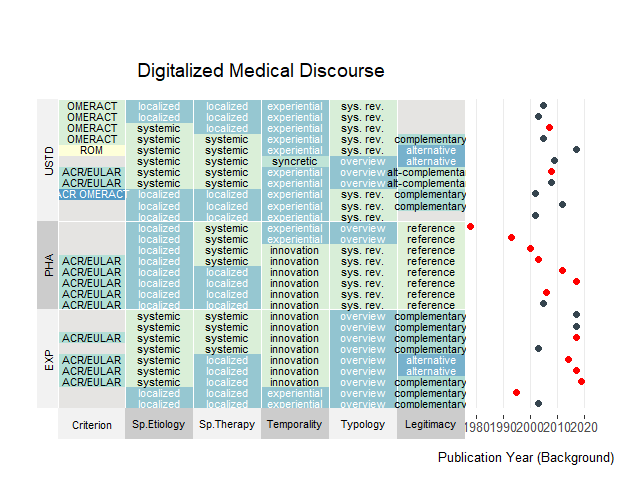


**Figure S1** *Representation of the digitalized medical discourse in a heatmap, emphasizing socio-anthropological variables, versus biomedical categories. Colors are only used to distinguish elements, with no other meaning, their values is directly labeled in the cells of the table. Light gray values are NA. Adjacent plot represents the corresponding Publication Year. Dots in red represent main authors’ Background in rheumatology, in black everything else. Geography is omitted given the relatively uninformative content (Eurocentric). The majority of Rheumatologists in PHA versus the two other categories is visually clear, as is the extent of the temporal articles distribution.*

**Bibliography**

1. Lundeberg T, Lund I, Sing A, Naslund J. Is placebo acupuncture what it is intended to be? Evidence-based complementary and alternative medicine : eCAM. 2011;2011: 932407. doi:10.1093/ecam/nep049

2. Lundeberg T, Lund I, Näslund J, Thomas M. The Emperors sham - wrong assumption that sham needling is sham. Acupunct Med. 2008;26: 239–242. doi:10.1136/aim.26.4.239

3. International Conference on Harmonisation of technical requirements for registration of pharmaceuticals for human use. ICH harmonized tripartite guideline: Guideline for Good Clinical Practice. J Postgrad Med. 2001;47: 45–50.

4. Casimiro L, Barnsley L, Brosseau L, Milne S, Robinson VA, Tugwell P, et al. Acupuncture and electroacupuncture for the treatment of rheumatoid arthritis. The Cochrane database of systematic reviews. 2005; Cd003788. doi:10.1002/14651858.CD003788.pub2

5. Lee MS, Shin B-C, Ernst E. Acupuncture for rheumatoid arthritis: a systematic review. Rheumatology (Oxford). 2008;47: 1747–1753. doi:10.1093/rheumatology/ken330

6. Wang C, de Pablo P, Chen X, Schmid C, McAlindon T. Acupuncture for pain relief in patients with rheumatoid arthritis: a systematic review. Arthritis Rheum. 2008;59: 1249–1256. doi:10.1002/art.24009

7. Hafferty F, Castellani B. MEDICAL SOCIOLOGY. 2019. pp. 331–338.

8. Cohn S, Lynch R. Diverse bodies: the challenge of new theoretical approaches to medical anthropology. Anthropology & Medicine. 2017;24: 131–141. doi:10.1080/13648470.2017.1334395

9. Clarke AE, Shim JK, Mamo L, Fosket JR, Fishman JR. Biomedicalization: Technoscientific Transformations of Health, Illness, and U.S. Biomedicine. American Sociological Review. 2003;68: 161–194. doi:10.2307/1519765

10. Fovargue S, Mullock A. The Legitimacy of Medical Treatment: What role for the medical exception. Taylor & Francis; 2016. Available: https://library.oapen.org/handle/20.500.12657/32737

11. Gale N. The Sociology of Traditional, Complementary and Alternative Medicine. Sociol Compass. 2014;8: 805–822. doi:10.1111/soc4.12182

12. Lambert H, Rose H. Misunderstanding science?: Disembodied knowledge? Making sense of medical science. 1996. doi:10.1017/CBO9780511563737.004

13. Coletti MH, Bleich HL. Medical Subject Headings Used to Search the Biomedical Literature. J Am Med Inform Assoc. 2001;8: 317–323.

14. Ganesan K, Gengadharan AC, Balachandran C, Manohar BM, Puvanakrishnan R. Low frequency pulsed electromagnetic field--a viable alternative therapy for arthritis. Indian J Exp Biol. 2009;47: 939–948.

15. Institute of Medicine (US) Committee on the Use of Complementary and Alternative Medicine by the American Public. Complementary and Alternative Medicine in the United States. National Academies Press (US). 2005.

16. Christie A, Jamtvedt G, Dahm KT, Moe RH, Haavardsholm EA, Hagen KB. Effectiveness of nonpharmacological and nonsurgical interventions for patients with rheumatoid arthritis: an overview of systematic reviews. Phys Ther. 2007;87: 1697–1715. doi:10.2522/ptj.20070039

17. Michel Foucault. The Birth of the Clinic: An Archaeology of Medical Perception. Routledge Taylor&Francis Group. 1963.

18. 9780691121918: The Politics of Life Itself: Biomedicine, Power, and Subjectivity in the Twenty-First Century (In-Formation) - AbeBooks - Rose, Nikolas: 0691121915. [cited 28 Feb 2022]. Available: https://www.abebooks.com/9780691121918/Politics-Life-Biomedicine-Power-Subjectivity-0691121915/plp

19. Aletaha D, Neogi T, Silman AJ, Funovits J, Felson DT, Bingham CO 3rd, et al. 2010 rheumatoid arthritis classification criteria: an American College of Rheumatology/European League Against Rheumatism collaborative initiative. Annals of the rheumatic diseases. 2010;69: 1580–8. doi:10.1136/ard.2010.138461

20. Prevoo MLL, Hof MA van ’t, Kuper HH, Leeuwen MA van, Putte LBA van de, Riel PLCM van. Modified disease activity scores that include twenty-eight-joint counts: development and validation in a prospective longitudinal study of patients with rheumatoid arthritis. 48. 1995 [cited 15 Mar 2022]. Available: https://repository.ubn.ru.nl/handle/2066/20651

21. Naredo E, Wakefield RJ, Iagnocco A, Terslev L, Filippucci E, Gandjbakhch F, et al. The OMERACT ultrasound task force--status and perspectives. The Journal of rheumatology. 2011;38: 2063–7. doi:10.3899/jrheum.110425

22. Nelson NL, Churilla JR. Massage Therapy for Pain and Function in Patients With Arthritis: A Systematic Review of Randomized Controlled Trials. Am J Phys Med Rehabil. 2017;96: 665–672. doi:10.1097/PHM.0000000000000712

23. Casimiro L, Brosseau L, Robinson V, Milne S, Judd M, Well G, et al. Therapeutic ultrasound for the treatment of rheumatoid arthritis. Cochrane Database Syst Rev. 2002; CD003787. doi:10.1002/14651858.CD003787
