# Supplementary material for: Semi-automated socio-anthropologic analysis of the medical discourse on rheumatoid arthritis: Potential impact on public health
Source: PLoS One. 2022 Dec 29;17(12):e0279632. doi: 10.1371/journal.pone.0279632 (PMC9799325; doi:10.1371/journal.pone.0279632)
Supplement: Attachment — Submitted filename: ResponseToReviewers.pdf [file pone.0279632.s005.pdf]

Reviewer #1: *The authors have presented their study related to semi-automated socio-anthropologic analysis of the medical discourse on Rheumatoid arthritis. The goal of the study is quite interesting and the results of the presented well. Some of the following concerns in the manuscript, if addressed could make the manuscript more consistent and further strengthen the author's conclusions:*

1. Technically, the abstract could be slightly revised and made more precise for the users to understand the overall study.

In the absence of specific requests we added subtitles clarifying the meaning of each step, hoping that this responds to the Reviewer's expectations

2. Introduction explains more insights related to the study but requires few revisions and changes. For Example sentences such as "biology and biochemistry to medical research or practice" [12], including by now bioinformatics, computational and systems biology [13] as well as network medicine [14,15], …" needs revision.

We have modified the sentence to make it more clear as follows:

"the investigation of pathological situations using biological theories and methods: the laws of the normal applied to pathological bodies" [12], *which includes nowadays modern disciplines like* bioinformatics, computational and systems biology [13] as well as network medicine [14,15]"

3. Minor changes such as terms "data base" needs to be changed as "database".

We changed as requested

4. Careful revision of manuscript is required for easy understanding of the readers.

We fulfilled point by point the specific requests of the two Reviewers and further revised the whole article thoroughly to make if clearer and easier to follow. In particular: we structured the Abstract, shortened the Introduction, enriched the Supplementary Information, reorganized and clarified all Materials and Methods, revised the Results, expanded the Conclusions.

5. In the methods section, kindly modify the following sub-headings:
"Materials – reviews, Materials – Variables, Methods Analysis."

We agree with the reviewer that this part may be confusing, breaking our approach into clearly labeled steps was among the most complex tasks in such an interdisciplinary approach. We therefore modified as follows:

We removed the "Material and Methods" heading, to keep:

- *Materials* which includes now the former “Material-reviews” renamed “*Data*” and the section *Variables*, which seems to us to well describe the associated section.
- *Methods* which now has three subheadings namely: *Digitalization*, *Textual Analysis*, *Automatic learning*

6. In methods section, describe the unsupervised learning technique used in this study. Techniques used in this study such as unsupervised clustering is not well explained in the manuscript. Only one sentence explains it, in the discussion part.

We thank the reviewer for highlighting this aspect, there is now a dedicated subheading, we have expanded the explanation in the methods part including an intuitive description before the more formal one, which now reads as follows:

“Second, to search for *emergent properties*, given the low dimensionality of the space and the dataset, statistical methods were not applicable. *Therefore, we chose a graphical approach to visualize up to six dimensions (variables) on the bidimensional space of the manuscript*. Intuitively, we positioned the articles represented as points in a six-dimensional space, and looked for areas where these points are denser. I.e. Patterns were searched by visual inspection of the clusters identified as areas *where items are closer among them than they are to other points in the space*. In particular, the items are dots represented by the variables’ graphical distributions on a scatter plot, mapping the six variables”

7. This study has certain limitations such as only 28 articles are used in the analysis both for textual and automated analysis. Can this approach be implemented for other studies with a large dataset (articles)?

We addressed the point about limitations also in point 2 of Reviewer 2, and moved this section to the Discussion part. We also added an embryo of the discussion that pertains to the *generalization* and *scalability* of this approach in the Conclusion: in short, yes, theoretically, the approach can be implemented for other studies (generalizability) with a larger dataset (scalability). Clearly, time is a limiting factor at this point, we therefore discuss the potential of automated methods’ training (structural topic modelling) based on the current work that we hope to be pioneering and promoter of future developments in this direction.

Search terms used for the article selection from PubMed can also be included in the methods section.

As mentioned above (see point 5) casting this interdisciplinary and original approach into the Material and Methods section according to 3 different disciplines was a tremendous challenge. We agree with the Reviewer that it would also be possible to move this into Methods, but we feel at the same time that Materials would probably appear unbalanced, we therefore resorted to the solution that you read.

8. Careful editing of the sentences such as
“modulators of the ANS, which coincides to date with vagus nerve stimulation (VNS), which jointly represent novel experimental therapies (EXP)”

We modified as follows:

“These therapies include: (i) modulators of the *GI microbiome*: diet, nutraceutics, antibiotics and fecal microbial transplant (FMT); (ii) modulators of the *ANS*, which coincides to date with vagus nerve stimulation (VNS). Both the latter represent novel experimental therapies (*EXP*). Finally, we included (iii) *wound healing* modulators in the form of optical, mechanical, magnetic and electrical stimulation-based therapeutic approaches, offering limited standardization, evidence-based research, guidelines, funding and overall public recognition, despite widespread use. We name the approaches falling under this third point *unstandardized non-pharmacological (USTD).”*

“In the following: variables represents also the dimensions of the socio-anthropological space, ..”
will make the manuscript more readable for the users.

We agree this requires a longer clarification, we therefore added two paragraphs, one in the text:

“Finally, few words are needed to clarify the mathematical jargon, necessary to explain and justify the automatic analysis: *variables* represents also the *dimensions* of the socio-anthropological space, and *values* of the categorical variables are interchangeably referred to as *levels.*

The dimensionality of the nominal levels (number of values that a given variable can take) has importance in the variables selection. When this dimensionality is high, comparable to the number of items, it become uninformative for automated analyses, intuitively, each value is (almost) unique to an item, therefore generalization, groupings and patterns cannot be identified. For this reason Table 1 discriminates the low and high levels’ dimensionality of the variables.”

And one in the caption of Table 1

“column #Levels points to the dimensionality of the variable values (i.e. how many levels a variable can take, it corresponds to the different values that can be observed in each column of Table 2), a criterion that guides the analysis presented in Results (textual or automated). “

We also carefully checked the whole text to clarify where needed statements whose complexity can be referred to the interdisciplinarity of the work (mathematical notations within socio anthropological discussion)

Reviewer #2: *The authors have presented a view interesting work on medical discourse related to rheumatoid arthritis. Their study is an interdisciplinary biomedical research work that need expertise in anthropology, sociology, and medicine.*

Major revisions
1. Introduction section is very long. Specifically, the authors have presented a detailed note on the three stages or parts of their work in the introduction section. Instead, they can provide a summary that will motivate the readers to look into the methods immediately.

We appreciate the recommendation of the Reviewer and therefore have modified the text by making the introduction more succinct, and shifting the longer description partly in Materials and partly in the Methods (Variables) as recommended.

2. The authors have provided the limitations of their work in the introduction itself. This has to be moved to discussion, probably with a sub-heading, "Limitations".

We have modified as requested

3. The definition for each variable is helpful. However, it could be short. A detailed description on each variable in the methods section may take the readers away from the main research information.

Following up from point 1, we moved the detailed description of the Methods in Supplementary Information, and left in the Methods the description that was originally in the Introduction (basically: we summarized information in the Introduction, then moved -after adaptation- the longer part from Introduction to Methods, then moved -after adaptation- the longer part of Methods to Supplementary Information).

4. The authors use only six variables for the automated learning (page 12). Why? A brief note in he manuscript will be useful.

We agree this requires clearer explanation, and thank the reviewer for pointing this out. In short, only these six variables have the dimensionality of levels (i.e. number of values that a variable can take) compatible with this type of analysis. Intuitively, if the number of values that a variable can take is comparable to the number of items under study, then this value of the variable resembles more to a unique ID (useless to identify patterns and similarities) than to a characteristic typical of the dataset. To clarify this concept we first detailed the meaning of level dimensionality (see the second part of point 8 of Reviewer 1) and why if a variable can assume a number of values that is comparable to the size of the dataset it becomes meaningless in pattern search. Second, and this addresses the current point of Reviewer 2, we detailed the reasons why we discarded from this analysis the 3 variables Year of Publication, Background, Geography as updated in the Methods Digitalization (levels dimensionality are a consequence of the process of digitalization).

"Two different approaches are needed owing to the informativeness that accompany variables characterized by high or low levels' dimensionality (see Materials Variables). When there are too many levels and uniformly distributed, it becomes for instance meaningless to work with frequencies (absolute majority) as no variable will dominate and all values will be NA, this is the case for the Year of Publication, Geography and Background which were therefore discarded from the automatic analysis. However, the historical progression of PHA, the exclusive eurocentricity of the scientific production and the almost perfect overlap between RA background and PHA are characteristic that deserve discussion in the textual socio-anthropological analysis."

5. The automated learning section need to be elaborated for better understanding of the method implemented by the authors. How did the authors implement the automated learning? Which package did they use?

We have expanded the section to better describe the two approaches, adding for each an intuitive explanation, before the more formal definition, and we have moved at the beginning of the section the information on the language (R) and packages (ggplot and superheat) used.

6. The results section must include the results or findings. Rest can go to the discussion section. The authors should try to present the results in Tables or Figures, not just the text (see the sub-heading, "Textual analysis – individual variables). The result section must be revised completely.

As we mentioned in points 5 and 7 of Reviewer 1, organization of the material in such an interdisciplinary approach was among the most difficult tasks of our work. Indeed, what are Materials for one discipline are already Results for another, the same holds for Discussion and Results and so on. We therefore had to make decisions that could accompany any (biomedical, socio-anthropologist) reader along our analysis.

Having said this, we integrated the Reviewer's observation with our considerations and modified as follows: Section Results is divided in 3 parts, each pertaining to the outcome of 3 different approaches (Methods) namely: Digitalization, socio-anthropological textual analysis, Automated learning.

We have therefore simplified, clarified and renamed both in Methods and in Results each section where the same subheading indicates clearly, in our intention, which result pertains to which analysis.

7. The authors must revise the sub-headings in such a way that the sub-heading provides or summarizes the content in the paragraphs within it.

We hope our explanations and implementations of all the points above solve this point as well

8. Why the authors performed supervised learning on some variables and unsupervised learning on other variables? What is the reason? Can they apply either one? What happens if the learning approaches are swapped?

We thank the reviewer for pointing to the fact that our explanation may be confusing. In fact both the supervised and unsupervised analysis are performed on the same 6 variables whose levels' dimensionality is appropriate for the automatic analysis, i.e. Typology, Temporality, Spatialization Etiology and Therapy, Criterion and Legitimacy. In fact, the two approaches enable two different types of queries. The supervised analysis tells us how the socio-anthropological variables describe three categories we care about i.e. PHA, EXP, USTD. In other words, this analysis let us describe PHA, EXP and USTD in terms of the 6 socio-anthropological variables. Conversely, the unsupervised analysis observes how the 6 variables level group together spontaneously in our data, i.e. whether the 6 variables can be used not

only to describe the three known categories PHA, EXP, USTD, but also new, yet unnamed categories. For instance one of the clusters in Quadrant II let us observe that there exists commonality between a subset of USTD (electrical and optical stimuli) and a PHA (antibiotics), all sharing coherent *local* spatialization, i.e. these are therapies that assume a local vision of the etiology and therapy of RA, which is in contrast with EXP, USTD using mechanic and magnetic stimuli and all other PHA perceptions which have at least one systemic dimension of the spatialization. This observation which would be difficult to observe by other means than the unsupervised approach, clearly shows for example how the "discourse" is impactful, given for example the fact that VNS, a promising EXP, uses indeed electric stimuli.

We have clarified this by adding a longer explanation in the Results Digitalization section

Minor revisions
1. "(level I) the modulators of the GIP [19]," should be "the modulators of the GIP [19] (level I),"

Corrected

2. The sub-heading "Materials – Variables" should be within "Materials and Methods". The font size must be similar to "Materials – Review".

We have reorganized these sections as Material separate from Methods, and checked size and hierarchy carefully and accordingly

3. The sub-headings with Materials and Methods (e.g. Materials – Variables, Materials - Review) could be renamed for better clarity.

See also point of Reviewer 1: they were renamed simply Variables and Data

4. In table 1, the variables under Low and High levels is unclear.

This was corrected, see second part of point 8 of reviewer 1 and your point 4

5. Methods Analysis could have a better name.

This, like many other was changed, see points above
