# Supplementary material for: Semi-automated socio-anthropologic analysis of the medical discourse on rheumatoid arthritis: Potential impact on public health
Source: PLoS One. 2022 Dec 29;17(12):e0279632. doi: 10.1371/journal.pone.0279632 (PMC9799325; doi:10.1371/journal.pone.0279632)
Supplement: Attachment — Submitted filename: Rebuttal.docx [file pone.0279632.s006.docx]

**Reviewer #1**: Overall, the content and language has been improved by the authors with several changes, but there are still issues with syntactical constructions and formal scientific writing (e.g. in the added/extended text sections). Modification of subtitles and its content is more helpful for the readers to gain better insight of your research. If the manuscript is finally accepted, it should be looked at carefully for language check and sentence simplifications.

*We carefully checked the language, with the kind help of a mother tongue colleague and simplified the sentences across the whole article*

Some of the minor corrections are as follows:
1. Abstract has Background followed by Aim. In my opinion, abstract does not require the ‘aim’ section. Usually background followed by methods, results and conclusion would make it.

*Section Aim was meant to highlight the limitation of the current situation (Background) and hence to justify the current work (Methods and Results). However, we finally merged the two sections, according to the request.*

2. Some sentences needs simplification for readers to understand, (For example: “Our aim with this study is to contribute from yet another viewpoint, i.e. by analyzing how NCDs’ management is currently conceived and presented, using as proxy to gain insight into this issue an interdisciplinary analysis of the medical discourse.”)

*We agree, we broke in two sentences here and checked the whole text*

3. Is ‘automated’ and ‘automatic’ learning the same in the manuscript. Kindly maintain the consistency throughout the manuscript.

*Done*

4. Does this sentence explain the methodology used or the manuscript?
“Therefore, we chose a graphical approach to visualize up to six dimensions (variables) on the bidimensional space of the manuscript”. Could this sentence be modified for more clarity.

*We meant to indicate that we were able to accommodate 6 variables in a bidimensional space (the page of an article) but we understand that this was unnecessary and confusing, we removed the second part of the sentence*

5. Conclusion section is quite long. If this could be precise, it would be appreciated.

*We have created very short subsections to highlight precisely the themes that in our opinion represent the conclusion and deserve attention, we hope this addresses the issue*

Reviewer #2: 1. The paragraph that starts as “First, our analysis is run from the biomedical standpoint,” in page 49 and the paragraph that starts as “An overview of our approach is shown in Fig 1.” In page 50 include text on the author’s approach. The content is too long as well as clarity is missing. In the introduction, the authors should try to highlight the advancements they made. Their approach or pipeline should be presented as a summary. It must be clear and easy to understand.

*We thank the reviewer for this comment and we further simplified the introduction, moving the description of the three labels (PHA, EXP; USTD) in the methods, and pointing directly at the summary description supported by Figure 1*

2. Fig 1 can be cited in the beginning of methods. An overview of the approach (if required) can be presented withing the methods section.

*See observation n.1, we believe a graphical representation of the method can be really helpful in the introduction, but moved the details in the methods section as requested*

3. Is the subheading “Variables” come under “Materials”? The font size shows that they both are independent subheadings. The authors should check the instructions on the subheadings allowed in the journal.

*Yes correct: Variables and Data have the same size (Arial 14pt) and are indeed subheadings of Materials (Arial 16pt)*

4. The authors should recheck the language in the entire manuscript. For example, “Unsupervised Second, to search for emergent properties” is not grammatically correct.

*Corrected*

5. The authors can’t give the same subheading names within Methods and Results.

*We specifically did this when addressing the previous round of comments to clarify which Results belong to which Method and enhance the clarity of the whole text. We may add the mention “methods” and “results” before each headings but we feel this would make the text unnecessarily redundant, given the sub-heading structure, but we can of course modify if requested. We nevertheless added a sentence at the beginning of the Results section to clarify this structure matching*.

6. The authors can present the results in a better way. Current version includes only text.

*We do not understand this comment, the Results section includes one figure per type of results (three sections, three types of results, one shown in a table and two with graphs), explained at length also in the caption and supported by Supplementary Material where needed, maybe we did not understand the request?*
